# Identifying Cell-Penetrating Peptides for Effectively Delivering Antimicrobial Molecules into *Streptococcus suis*

**DOI:** 10.3390/antibiotics13080725

**Published:** 2024-08-02

**Authors:** Jinlu Zhu, Zijing Liang, Huochun Yao, Zongfu Wu

**Affiliations:** 1 MOE Joint International Research Laboratory of Animal Health and Food Safety, College of Veterinary Medicine, Nanjing Agricultural University, Nanjing 210014, China; 2021207067@stu.njau.edu.cn (J.Z.); 2019207013@stu.njau.edu.cn (Z.L.); yaohch@njau.edu.cn (H.Y.); 2 Key Lab of Animal Bacteriology, Ministry of Agriculture, Nanjing 210014, China; 3 WOAH Reference Lab for Swine Streptococcosis, Nanjing 210014, China; 4 Guangdong Provincial Key Laboratory of Research on the Technology of Pig-Breeding and Pig-Disease Prevention, Guangzhou 511400, China

**Keywords:** cell-penetrating peptides, antisense oligonucleotides, peptide nucleic acids, *Streptococcus suis*

## Abstract

Cell-penetrating peptides (CPPs) are promising carriers to effectively transport antisense oligonucleotides (ASOs), including peptide nucleic acids (PNAs), into bacterial cells to combat multidrug-resistant bacterial infections, demonstrating significant therapeutic potential. *Streptococcus suis*, a Gram-positive bacterium, is a major bacterial pathogen in pigs and an emerging zoonotic pathogen. In this study, through the combination of super-resolution structured illumination microscopy (SR-SIM), flow cytometry analysis, and toxicity analysis assays, we investigated the suitability of four CPPs for delivering PNAs into *S. suis* cells: HIV-1 TAT efficiently penetrated *S. suis* cells with low toxicity against *S. suis*; (RXR)_4_XB had high penetration efficiency with inherent toxicity against *S. suis*; (KFF)_3_K showed lower penetration efficiency than HIV-1 TAT and (RXR)_4_XB; K8 failed to penetrate *S. suis* cells. HIV-1 TAT-conjugated PNA specific for the essential gyrase A subunit gene (TAT-anti-*gyrA* PNA) effectively inhibited the growth of *S. suis*. TAT-anti-*gyrA* PNA exhibited a significant bactericidal effect on serotypes 2, 4, 5, 7, and 9 strains of *S. suis*, which are known to cause human infections. Our study demonstrates the potential of CPP-ASO conjugates as new antimicrobial compounds for combating *S. suis* infections. Furthermore, our findings demonstrate that applying SR-SIM and flow cytometry analysis provides a convenient, intuitive, and cost-effective approach to identifying suitable CPPs for delivering cargo molecules into bacterial cells.

## 1. Introduction

Cell-penetrating peptides (CPPs) are a family of short peptides typically consisting of 4–40 amino acids that can cross cell membranes [1,2,3]. They have emerged as a promising tool for delivering various types of cargo, including proteins, peptides, nucleic acids, small-molecule drugs, and antisense oligonucleotides (ASOs), into eukaryotic cells and bacteria due to their high transduction efficiency and low cytotoxicity [4,5,6,7,8]. In recent years, there has been a growing interest in exploring CPPs for delivering ASOs, including peptide nucleic acids (PNAs) and phosphorodiamidate morpholino oligomers (PMOs), for combating multidrug-resistant bacterial infections [9,10,11]. These ASOs, as antimicrobial molecules, hybridize with mRNA from essential species-specific bacterial genes at translation initiation regions, employing translation inhibition to preferentially kill bacterial pathogens while sparing the natural bacterial flora [8,12,13]. Moreover, ASOs can be easily modified to target evolving mutations and genetic changes [14,15]. Based on these features, CPP-ASO conjugates have emerged as promising species-specific antisense antimicrobials with low cytotoxicity [16,17], demonstrating efficacy in vitro and in vivo against various bacterial species by targeting essential genes required for bacterial growth such as *gyrA* [18,19], *acpP* [11,17,20], *ftsZ* [21], and *rpoA* [22]. However, the penetration efficiencies of CPPs as carriers vary depending on targeted bacterial species [18,23,24]. Moreover, there is a lack of convenient, intuitive, and cost-effective methods for screening CPPs with high penetration efficiency.

*Streptococcus suis*, a Gram-positive bacterium, is an important pathogen in the pig industry, causing septicemia, meningitis, and sudden death in pigs, resulting in substantial economic losses, and it is also an emerging zoonotic pathogen [25,26]. Based on the variation in capsular polysaccharide antigenicity, *S. suis* can be classified into 29 serotypes (1–19, 21, 23–25, 27–31, and 1/2) [27]. To date, 11 serotypes have been documented as capable of causing human infections, consisting of serotypes 1, 2, 4, 5, 7, 9, 14, 16, 21, 24, and 31 [26,28,29,30]. Among those serotypes, serotype 2 is widely recognized as the most prevalent and pathogenic serotype in both swine and humans worldwide [26,27]. Antimicrobial drugs have been employed as therapeutic and preventive medications for *S. suis* infection. However, the imprudent use of these drugs hastens the development of antimicrobial resistance, resulting in a severe multidrug-resistant state within *S. suis* [31,32,33,34]. The emergence of multidrug-resistant strains of *S. suis* is increasing at an alarming rate, calling for the urgent need to develop new antibacterial compounds. Thus, finding suitable CPPs to deliver antimicrobial molecules directly into *S. suis* cells is essential.

This study investigated the feasibility of delivering PNAs into *S. suis* cells using four CPPs, consisting of K8, (KFF)_3_K, HIV-1 TAT, and (RXR)_4_XB, which have previously been tested as carrier molecules in eukaryotic cells and are known to exhibit low toxicity [18,35,36,37]. We evaluated the penetration efficiency and toxicity of these peptides and determined the antibacterial effect of CPP-PNA conjugates in five serotypes of *S. suis* known to cause human infections. Our study shows CPP-PNA conjugates as promising new antimicrobial compounds against *S. suis* infections and provides a convenient, intuitive, and cost-effective reference method for screening suitable CPPs to deliver cargo molecules into bacterial cells.

## 2. Results

### 2.1. CPPs Penetration into S. suis

**Figure 1:**
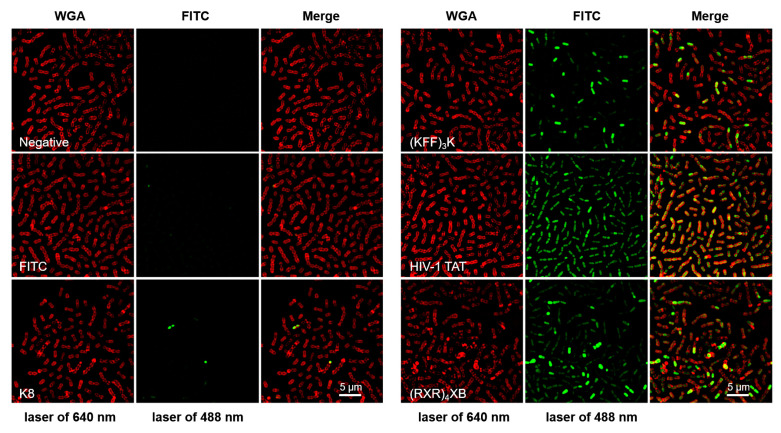
Representative images were used for analyzing the internalization efficiency of FITC-labeled CPPs in *S. suis* by SR-SIM. The negative control received an equivalent volume of water, while an equivalent concentration of FITC single molecules was also included as a control. The *S. suis* cell membrane was counterstained with Alexa Fluor 633-WGA (wheat germ agglutinin) and observed with a laser at a wavelength of 640 nm (WGA, red), while the fluorescence signal of FITC was observed using a laser at a wavelength of 488 nm (FITC, green).

To determine whether the CPPs K8, (KFF)_3_K, HIV-1 TAT, and (RXR)_4_XB listed in Table 1 can enter *S. suis* cells, we initially utilized super-resolution structured illumination microscopy (SR-SIM) to observe the intracellular localization of FITC-labeled CPPs in the *S. suis* cells. SR-SIM achieves higher spatial resolution than traditional microscopes through structured illumination patterns, allowing for the observation of smaller-scale bacterial structures and details. As shown in Figure 1, the *S. suis* cell membrane and FITC-labeled CPPs were observed with lasers at 640 nm (WGA, red) and 488 nm (FITC, green), respectively. Upon combined observation of WGA and FITC fluorescence signals, SIM images demonstrated that peptides HIV-1 TAT and (RXR)_4_XB at a concentration of 10 µM were able to efficiently cross the *S. suis* cell membrane and distribute within the cells (Merge). In contrast, (KFF)_3_K shows lower penetration efficiency than HIV-1 TAT and (RXR)_4_XB, while K8 fails to penetrate *S. suis* cells. However, we observed that (RXR)_4_XB appeared to possess a membrane-disruptive effect on *S. suis*, leading to a substantial accumulation of FITC-labeled (RXR)_4_XB within the cells. In contrast, HIV-1 TAT exhibited a more even intracellular distribution.

### 2.2. CPPs Uptake Efficiency Analysis by Flow Cytometry

**Figure 2:**
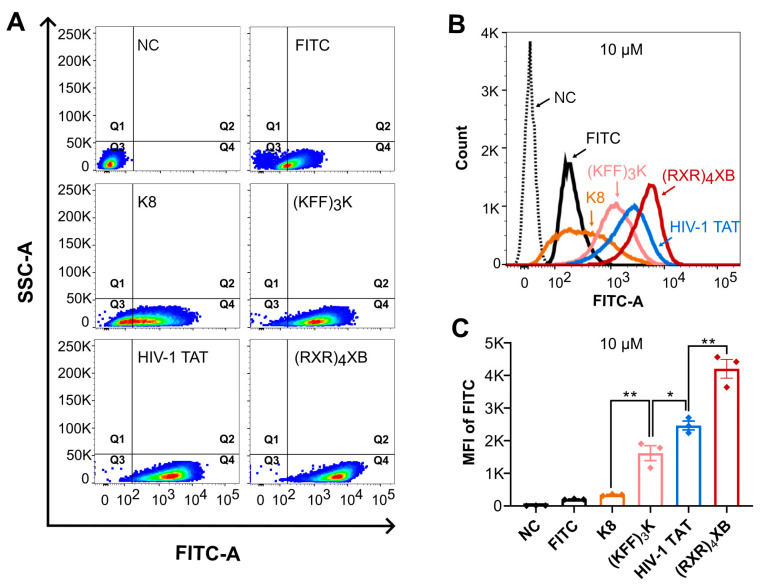
Cellular uptake of CPPs in *S. suis* was analyzed by flow cytometry. *S. suis* cells were exposed to 10 µM FITC-labeled CPPs, with fluorescence measured one hour post-treatment. The negative control (NC) received an equivalent volume of water, while an equivalent concentration of FITC single molecules was also included as a control. A total of 50,000 events were collected during the flow cytometry analysis. Dot plots (**A**) and histograms (**B**) were analyzed using FlowJo™ v10 software, with quadrant 3 representing the negative confidence region. SSC, or cell count, was plotted on the *y*-axis, and FITC fluorescence intensity was plotted on the *x*-axis. The MFI of FITC was analyzed using FlowJo™ v10 software (**C**). The unpaired *t*-test was used to compare the MFI of *S. suis*. * indicates *p* < 0.05, ** indicates *p* < 0.01.

To assess the uptake efficiency of K8, (KFF)_3_K, HIV-1 TAT, and (RXR)_4_XB in *S. suis* cells, we exposed *S. suis* serotype 2 strain SC070731 cells to 10 µM FITC-labeled CPPs and measured fluorescence using flow cytometry after one hour of treatment. A total of 50,000 events were collected, and over 95% of bacterial cells were acquired and analyzed by gating on side scatter (SSC) and forward scatter (FSC). As shown in Figure 2A, bacterial cells exhibited varying fluorescence intensities after treatment with different CPPs. The baseline fluorescence threshold of bacterial cells was determined by the negative control (water) and set as the negative confidence area (quadrant 3). *S. suis* cells treated with 10 µM FITC molecule, K8, (KFF)_3_K, HIV-1 TAT, or (RXR)_4_XB mainly clustered in quadrant 4, accounting for 64.80%, 71.20%, 98.90%, 99.80%, and 99.90% of total cells, respectively (Figure 2A). As shown in Figure 2B, *S. suis* cells treated with (KFF)_3_K, HIV-1 TAT, or (RXR)_4_XB exhibited an unimodal distribution in the histogram. The peaks of (RXR)_4_XB, HIV-1 TAT, and (KFF)_3_K were shifted to the right compared to those of the negative control and FITC molecule. The peak of (RXR)_4_XB was positioned furthest to the right, suggesting the most vigorous fluorescence intensity, followed by HIV-1 TAT and (KFF)_3_K. In contrast, K8 exhibited the weakest fluorescence intensity. We analyzed the mean fluorescence intensity (MFI) of *S. suis* cells using FlowJo^TM^ v10 software. Significant differences existed in the MFI of *S. suis* cells treated with 10 µM of different FITC-labeled CPPs. We found that (RXR)_4_XB exhibited the highest MFI, followed by HIV-1 TAT. In contrast, (KFF)_3_K showed a lower MFI than HIV-1 TAT, while K8 possessed the lowest MFI (Figure 2C). The results from flow cytometric analysis concurred with the observations from SR-SIM. These findings indicate that (RXR)_4_XB and HIV-1 TAT can efficiently penetrate *S. suis* cells. However, (KFF)_3_K displayed a lower penetration efficiency than (RXR)_4_XB and HIV-1 TAT, while K8 displayed a lowest penetration efficiency among them. Notably, the MFI of (RXR)_4_XB was significantly higher than that of HIV-1 TAT. This phenomenon might be associated with the potential cell membrane disruption effect of (RXR)_4_XB on *S. suis* cells observed by SR-SIM. To select suitable CPPs, we must balance their cell penetration efficiency and toxicity to target cells. Therefore, for CPPs with high penetration efficiency, further analysis of their toxicity to target cells is crucial.

### 2.3. Toxicity Analysis of HIV-1 TAT and (RXR)_4_XB

**Figure 3:**
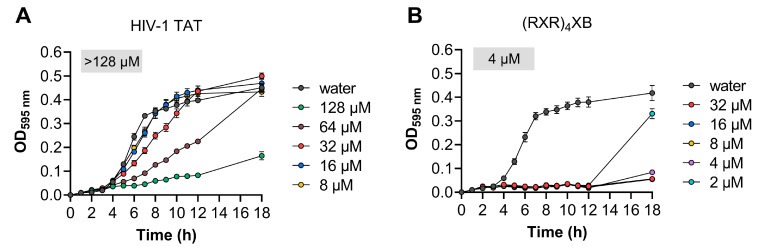
Growth kinetics and MIC determination for *S. suis* serotype 2 strain SC070731 in various concentrations of CPPs. (**A**) HIV-1 TAT at concentrations ranging from 128 to 8 µM. (**B**) (RXR)_4_XB at concentrations ranging from 32 to 2 µM. An equivalent volume of water was included as a control. The MIC value is shown and was determined as the lowest concentration inhibiting visible growth in the wells (OD_595nm_ < 0.1).

We determined the minimum inhibitory concentrations (MICs) of HIV-1 TAT and (RXR)_4_XB, which exhibited high penetration efficiency in *S. suis* cells, using *S. suis* serotype 2 strain SC070731. We found that HIV-1 TAT did not induce significant growth inhibition in SC070731 (MIC > 128 µM; Figure 3A). However, (RXR)_4_XB resulted in growth inhibition (MIC of 4 µM; Figure 3B). We investigated the concentration-dependent bactericidal activity of HIV-1 TAT or (RXR)_4_XB by treating *S. suis* serotype 2 strain SC070731 with concentrations ranging from 8 to 64 µM. The results revealed no reduction in bacterial counts after HIV-1 TAT incubation within the 8 to 64 µM range. Conversely, a significant decrease in bacterial counts was observed after (RXR)_4_XB incubation within the 8 to 64 µM range (Table 2). Thus, (RXR)_4_XB possesses inherent toxicity against *S. suis*, whereas HIV-1 TAT is not.

### 2.4. HIV-1 TAT-Coupled gyrA-Specific PNA Exhibits Bactericidal Activity

**Figure 4:**
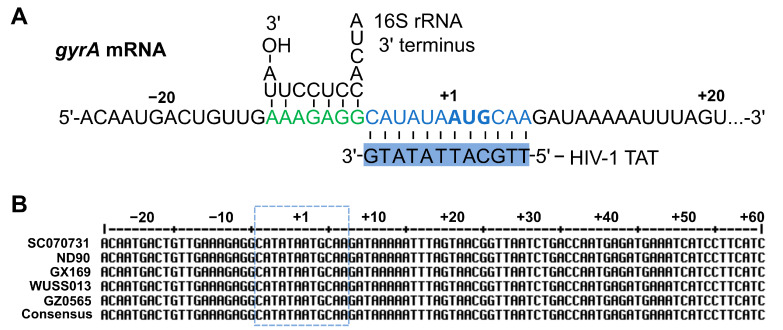
Schematic illustration of the PNA target region of gene *gyrA*. (**A**) Region of *gyrA* mRNA in *S. suis*, with the start codon (AUG) shown in bold type. For the location relative to the start site, ‘A’ of AUG is defined as +1 in this study. The Shine–Dalgarno and PNA target sequences are shaded in green and blue, respectively. Below, the PNA sequence is shown (blue box) with the conjugated CPP HIV-1 TAT for delivery into *S. suis*. (**B**) Multiple sequence alignments of the gene *gyrA* in different serotypes of *S. suis*, including serotype 2 strain SC070731, serotype 4 strain ND90, serotype 5 strain GX169, serotype 7 strain WUSS013, and serotype 9 strain GZ0565. A defined section (−26 to +60 nt), including the region around the PNA binding site (blue dashed border), is shown.

To assess the capability of HIV-1 TAT to deliver cargo molecules into *S. suis*, we utilized a widely used *gyrA*-specific antisense PNA as the delivered molecule. The *gyrA*-specific antisense PNA fully complements the 12 nucleotides surrounding the *gyrA* mRNA start codon (Figure 4A), a sequence highly conserved among different serotypes of *S. suis* (Figure 4B). HIV-1 TAT is the carrier, conjugated to the 5′ end of the *gyrA*-specific antisense PNA via the linker 8-amino-3,6-dioxaoctanoic acid.

**Figure 5:**
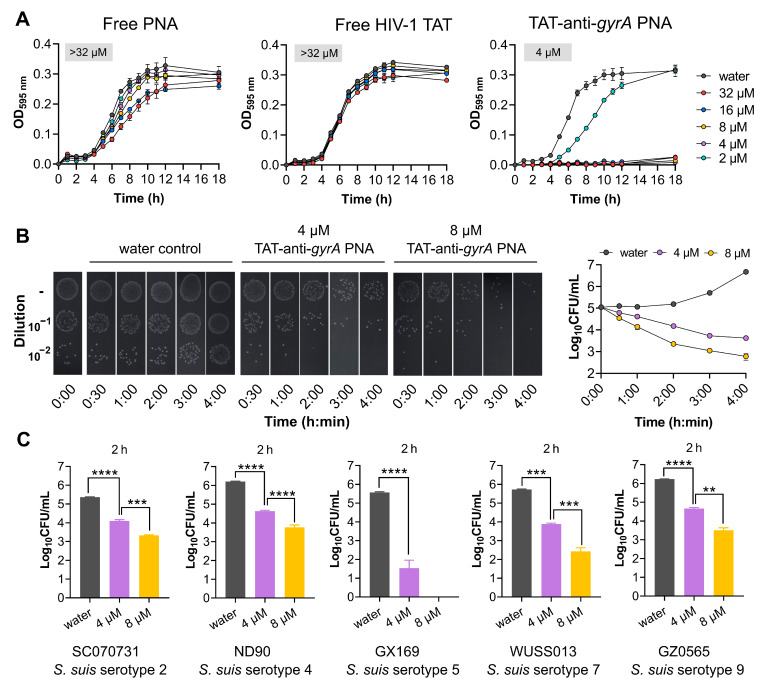
An antisense *gyrA*-specific PNA coupled to HIV-1 TAT (TAT-anti-*gyrA* PNA) exhibits antibacterial activity against *S. suis* strains. (**A**) Growth kinetics and MIC determination of SC070731 in various concentrations of free *gyrA*-specific PNA, free HIV-1 TAT, or TAT-anti-*gyrA* PNA at concentrations ranging from 32 to 2 µM. The MIC value is shown and was determined as the lowest concentration inhibiting visible growth in the wells (OD_595nm_ < 0.1). (**B**) Bactericidal effects of TAT-anti-*gyrA* PNA were determined at 1 × MIC (4 µM) and 2 × MIC (8 µM) against *S. suis* serotype 2 strain SC070731 during a 4-h time course. After the indicated time points, aliquots of each condition were subjected to spot assays or CFU determination on THA plates to investigate the number of viable cells. (**C**) Concentration-dependent reduction in the bacterial counts following treatment of *S. suis* strains of various serotypes with TAT-anti-*gyrA* PNA for 2 h. The unpaired *t*-test was used to compare the number of viable bacterial cells. ** indicates *p* < 0.01, *** indicates *p* < 0.001, **** indicates *p* < 0.0001.

The effectiveness of HIV-1 TAT as a PNA carrier targeting *S. suis* was assessed by evaluating the bactericidal activity. The results revealed that free peptide or PNAs controls did not induce significant growth inhibition in *S. suis* serotype 2 strain SC070731 (MIC > 32 µM; Figure 5A). However, HIV-1 TAT-coupled *gyrA*-specific PNA (TAT-anti-*gyrA* PNA) led to efficient growth inhibition (MIC of 4 µM; Figure 5A). To clarify whether the observed growth inhibition of TAT-anti-*gyrA* PNA on *S. suis* serotype 2 strain SC070731 is bacteriostatic or bactericidal, we conducted a 4-h bactericidal assay using 1 × MIC and 2 × MIC concentrations against 1 × 10^5^ CFU/mL of bacteria. As shown in Figure 5B, colony-forming units (CFU) counting on THA plates revealed that 4 µM and 8 µM of TAT-anti-*gyrA* PNA significantly reduced CFU counts, with a noticeable decrease observed as early as 30 min post-treatment. As shown in Figure 5C, a significant reduction in bacterial numbers was observed for serotypes 2, 4, 5, 7, and 9 strains of *S. suis* exposed to TAT-anti-*gyrA* PNA after 2 h, indicating a bactericidal effect of TAT-anti-*gyrA* PNA on various serotypes of *S. suis*.

## 3. Discussion

ASOs, primarily comprising antisense PNAs and PMOs, are a type of synthetic non-cytotoxic nucleic acid derivative known as antisense antimicrobial molecules [8,38]. PNAs, constructed by attaching nucleobases to a modified polyamide backbone, are resistant to nucleases and proteases, maintaining high stability in complex biological environments, including human serum and cell extracts [13,39]. Their uncharged nature contributes to their high affinity for mRNA [13]. Upon delivery to the cellular target, they can leverage Watson–Crick nucleic acid base pairing to function as translation inhibitors by sterically hindering the ribosome-mRNA interaction, thereby silencing the expression of specific genes [8,13]. However, due to the selective permeability of cell membranes, PNAs are usually unable to enter bacterial cells on their own. Therefore, PNAs are typically conjugated to CPPs to facilitate uptake into bacterial cells [8,40]. Several studies have reported that the essential *gyrA* gene has been successfully targeted using PNAs delivered by CPPs such as K8, (KFF)_3_K, HIV-1 TAT, and (RXR)_4_XB, demonstrating antibacterial effects in both Gram-positive and Gram-negative organisms. (KFF)_3_K-coupled *gyrA*-specific PNAs have shown antimicrobial effects in *S. pyogenes* [18,19], *S. aureus* [41,42], and *A. baumannii* [43]; HIV-1 TAT and (RXR)_4_XB have been identified as valuable carriers for *gyrA*-specific PNAs in *S. pyogenes* [18,19] and *S. pneumoniae* [9]; K8-coupled *gyrA*-specific PNAs have shown antimicrobial effects in *S. pyogenes* but did not demonstrate bactericidal activity in *S. pneumoniae* [9,18]. Various CPPs demonstrate different efficiencies in delivering PNAs, and the same CPPs may exhibit varying delivery efficiencies in different bacterial species. To achieve the optimal antibacterial effect of CPP-ASO conjugates, selecting suitable CPPs specific to the targeted bacterial species is crucial.

Previous research has indicated that arginine-rich polymers exhibit significantly enhanced cellular uptake compared to polymers of similar length containing lysine [44]. This study demonstrated the efficient penetration of *S. suis* cells by arginine-rich CPPs HIV-1 TAT and (RXR)_4_XB. In contrast, (KFF)_3_K shows lower penetration efficiency than HIV-1 TAT and (RXR)_4_XB, while K8 fails to penetrate *S. suis* cells. Although (RXR)_4_XB demonstrates high efficiency in penetrating *S. suis* cells, it exerts considerable toxicity towards the bacteria. The length of peptides is a crucial factor influencing their cellular uptake capability. Mitchell *et al*. reported that the penetration efficiency of arginine-rich polymers with fewer than five amino acids is notably lower than that of peptides containing six or more amino acids [44]. As the peptide length extends to 15 amino acids, penetration ability increases; however, cell toxicity rises concurrently [44]. The toxicity exhibited by (RXR)_4_XB in *S. suis* may be associated with its length. CPPs as delivery vectors should ideally not exhibit toxicity towards target bacterial cells [40]. The current primary research approach involves selecting various CPPs to conjugate with ASOs, evaluating their antibacterial efficacy by determining the MIC values of CPP-ASO conjugates, and assessing the ability of the chosen CPPs to deliver antimicrobial agents to specific bacterial species [18,20,45]. While this method is effective in screening CPP-ASO conjugates with antibacterial effects, it requires the evaluation of a significant number of conjugates. It may even require the conjugation of peptides with ASOs targeting different genes, thereby significantly amplifying the complexity and cost of the screening process. Here, by combining SR-SIM, flow cytometry, and toxicity analysis assays, we determined that HIV-1 TAT efficiently penetrates *S. suis* cells with low toxicity. HIV-1 TAT-conjugated PNA specific for *gyrA* effectively inhibited the growth of *S. suis*. Our findings demonstrate that CPP-PNA conjugates can be promising new antimicrobial compounds against *S. suis* infections and offer convenient, intuitive, and cost-effective methods for screening suitable CPPs to deliver cargo molecules into targeted bacterial cells.

Another innovative synthetic ASO strategy is targeting non-essential genes, such as genes involved in antibiotic resistance and virulence [13]. The strategy of targeting antibiotic resistance genes aims to decrease the expression of antibiotic resistance, thereby restoring susceptibility to an approved antibiotic that would be co-administered with the oligomer. Recently, it has been shown in various bacterial species that utilizing CPP-ASO conjugates to target antibiotic resistance genes can reinstate the susceptibility of resistant strains to standard antibiotics [46,47,48,49]. Moreover, suppressing the expression of virulence genes is regarded as a promising alternative antibacterial therapy compared to conventional methods [50,51,52]. This method aims to inhibit the expression of crucial virulence factors in pathogens, making them more susceptible to clearance by the host immune system. Furthermore, anti-virulence agents impose less selective pressure on the pathogen, thus decreasing the emergence of drug-resistant mutants compared to traditional antimicrobial drugs [50,52,53,54].

In addition to ASOs, CPPs have great potential for delivering various cargo molecules, including peptides and foreign DNA, to bacterial cells. In a recent study, Islam et al. demonstrated the transfer of large plasmid DNA into *Escherichia coli* cells through CPP-mediated delivery [55]. This delivery method is recognized for safeguarding the integrity of nucleic acid and is considered suitable for transporting large nucleic acid molecules [56]. Although electroporation and pheromone-induced natural transformation were used to introduce foreign DNA into *S. suis* cells [57,58], for some *S. suis* clinical isolates, it remains challenging to introduce foreign DNA into cells. The possibility of using CPPs to introduce foreign DNA into *S. suis* deserves further investigation.

In conclusion, our study has shown the potential of HIV-1 TAT as a delivery vehicle for antimicrobial molecules into *S. suis* cells and has demonstrated that using CPP-ASO conjugates represents a promising approach for combating *S. suis* infections. Future research should investigate more potential CPPs and target genes to identify antisense antimicrobial compounds with high antibacterial activity against *S. suis*. Additionally, our results underscore that SR-SIM and flow cytometry analysis serve as valuable tools for assessing the penetration efficiency of CPPs in *S. suis*, providing a convenient, intuitive, and cost-effective reference method for screening suitable CPPs with high penetration efficiency to deliver cargo molecules into bacteria.

## 4. Materials and Methods

### 4.1. Bacterial Strains and Culture Conditions

The following *S. suis* strains were used in the study: serotype 2 virulent strain SC070731, isolated from a pig with meningitis [59]; serotype 4 virulent strain ND90, isolated from a diseased pig [28]; serotype 5 strain GX169, isolated from a human patient and kindly provided by Dr. Han Zheng, Chinese Center for Disease Control and Prevention, China; serotype 7 virulent strain WUSS013, isolated from a diseased pig [30]; serotype 9 virulent strain GZ0565, isolated from a pig with meningitis [60]. All strains were streaked on Todd–Hewitt agar (THA) containing 5% (*v*/*v*) sheep blood and cultured in Todd–Hewitt broth (THB) at 37 °C with 5% CO_2_.

### 4.2. CPPs Synthesis and Fluorescent Labeling

All peptides in this study, listed in Table 1, were synthesized and purified using high-performance liquid chromatography (HPLC purity ≥ 95%) provided by GenScript Inc. (Nanjing, China). A fluorescein moiety (FITC) was attached to the N-terminus of peptides via a 6-aminohexanoic acid spacer (GenScript Inc.), and all FITC-labeled peptides were dissolved in ultrapure water.

### 4.3. SR-SIM Analysis

*S. suis* serotype 2 strain SC070731 was streaked onto THA plates and incubated at 37 °C with 5% CO_2_ for 12 h. A bacterial colony was picked and inoculated overnight in 2 mL of THB at 37 °C. The overnight culture was then diluted 100-fold in fresh THB and grown to an OD_600nm_ of 0.6 (approximately 3 × 10^8^ CFU/mL). The obtained culture was washed twice with PBS and resuspended in PBS. Further, 900 µL of the bacterial solution was transferred into a 2 mL tube. Immediately, 100 µL of a 10× FITC-labeled CPPs working solution was added to a final concentration of 10 µM. Controls were prepared using an equivalent volume of water or a 10× FITC molecule solution. The reaction tubes were incubated for one hour at 37 °C and 10 rpm rotation in the dark. The bacterial cells were washed twice with PBS, fixed with 4% paraformaldehyde for 20 min, and washed again before resuspending in PBS. They were then incubated with 5 µg/mL Alexa Fluor 633-WGA (wheat germ agglutinin) purchased from Thermo Fisher for 30 min at room temperature in the dark. The cells were then washed twice and resuspended in PBS. Subsequently, 3 µL of bacterial solution was applied onto a microscope slide and dried using an alcohol lamp. A droplet of ProLong^TM^ Diamond antifade mountant (Thermo Fisher, Waltham, MA, USA) was added next. The microscope slide was then covered with a cover slip, and fluorescent cells were observed using SR-SIM (Nikon, Tokyo, Japan). The fluorescence emitted by the 640 nm laser was used to analyze the cell membrane stained with Alexa Fluor 633-WGA. In contrast, the fluorescence emitted by the 488 nm laser was used to analyze the distribution of FITC-labeled CPPs in bacterial cells. Each experiment has been performed on at least three independent biological replicates.

### 4.4. Flow Cytometry Analysis

As mentioned, the bacterial cells were treated with FITC-labeled CPPs and fixed with 4% paraformaldehyde. To analyze the cell membrane penetration ability of the CPPs, flow cytometry analysis was performed using a FACSVerse^TM^ flow cytometer (BD Biosciences, Franklin Lakes, NJ, USA) equipped with a 488 nm laser for FITC detection. Bacterial cells in suspension were analyzed at a 200–300 events/s flow rate, and 50,000 events were recorded for analysis. The acquisition and analysis were performed by gating on side scatter (SSC) and forward scatter (FSC). The mean fluorescence intensity of FITC was analyzed using FlowJo^TM^ v10 software (BD Biosciences). Each experiment has been performed on at least three independent biological replicates.

### 4.5. Synthesis of PNAs and CPP-PNA Conjugates

PNAs and CPP-PNA conjugates, as listed in Table 1, were obtained from PANAGENE Inc. (Daejeon, Republic of Korea). The PNAs contained a sequence complementary to the mRNA of the target gene for the gyrase A subunit (*gyrA*), a cellular target for quinolone antibiotics [61]. The peptide HIV-1 TAT was attached to *gyrA*-specific PNAs via the linker 8-amino-3,6-dioxaoctanoic acid. The quality and purity of these constructs were confirmed to be sufficient through HPLC analysis (purity > 99%) and mass spectrometry. The PNAs and CPP-PNA conjugates were dissolved in ultrapure water. The absorbance at 260 nm of these solutions was measured using a NanoDrop spectrophotometer, and the concentrations were then calculated using the extinction coefficient based on recently published methods [23,62].

### 4.6. MIC Determination

The broth microdilution method was employed to determine the MIC values, following a previous study and a recently published protocol with a few modifications [23,62]. In brief, an overnight *S. suis* serotype 2 strain SC070731 culture was diluted 100-fold in fresh THB and grown to an OD_600nm_ of 0.6. Subsequently, the bacterial suspension was further diluted 2000-fold in fresh Mueller–Hinton broth (MHB) to a final concentration of approximately 1 × 10^5^ CFU/mL. Then, 180 µL of the bacterial solution was transferred into a 96-well plate. Immediately, 20 µL of a 10× peptides, PNAs, or CPP-PNA conjugates working solution was added. As a negative control, an equivalent volume of water was added instead. The plate was then incubated at 37 °C with 5% CO_2_ for 18 h, and the growth was periodically monitored by measuring the OD_595nm_ every hour using a BioTek ELx800 plate reader. The MIC value was determined as the lowest concentration inhibiting visible growth in the wells (OD_595nm_ < 0.1).

### 4.7. Determination of Bactericidal Effects

The bactericidal effects were determined according to a previous study, with a few modifications [23]. Overnight cultures of the respective *S. suis* strain were diluted 100-fold in fresh THB and grown to an OD_600nm_ of 0.6. Afterward, the bacterial suspension was further diluted 2000-fold to obtain a final concentration of 1 × 10^5^ CFU/mL in THB. Subsequently, appropriate dilutions were prepared and plated on THA plates to serve as the input condition for determining the number of colony-forming units (CFU). Simultaneously, 10 µL of this diluted bacterial culture and serial dilutions (10^−1^, 10^−2^, 10^−3^) were directly spotted onto THA plates. Further, 180 µL of the bacterial solution was transferred into a 2 mL tube. Immediately, 20 µL of 10× peptides or CPP-PNA conjugates working solution was added. As a negative control, an equivalent volume of water was added instead. The tube was incubated at 37 °C, and viable cell count determination and spot assay were performed at indicated time points after the treatment.

## Figures and Tables

**Table 1:** The list of CPPs, PNAs, CPP-PNA conjugates, and their MIC values for strain SC070731.

Name	CPP Sequence	PNA Sequence	MIC (µM)	Reference
K8	KKKKKKKK-NH_2_	-	-	[18]
(KFF)_3_K	KFFKFFKFFK-NH_2_	-	-	[35]
HIV-1 TAT	GRKKRRQRRRYK-NH_2_	-	>128	[36]
(RXR)_4_XB	RXRRXRRXRRXRXB-NH_2_	-	4	[37]
Free PNA	-	ttgcattatatg	>32	
TAT-anti-*gyrA* PNA	GRKKRRQRRRYK	ttgcattatatg	4	

CPP, cell-penetrating peptide; PNA, peptide nucleic acid. CPP sequences are represented in N- to C-terminal orientation. PNA sequences are represented in 5′ to 3′ terminal orientation. The CPP is conjugated to the 5′ end of the PNA via the linker 8-amino-3,6-dioxaoctanoic acid. K = Lysine; F = Phenylalanine; G = Glycine; R = Arginine; Q = Glutamine; Y = Tyrosine; X = 6-aminohexanoic acid; B = β-alanine.

**Table 2:** Concentration-dependent effects of HIV-1 TAT or (RXR)_4_XB on strain SC070731.

Treatment	8 µM		16 µM		32 µM		64 µM	
	(Lg CFU) ± SD	Lg CFU Reduction	(Lg CFU) ± SD	Lg CFU Reduction	(Lg CFU) ± SD	Lg CFU Reduction	(Lg CFU) ± SD	Lg CFU Reduction
HIV-1 TAT	6.37 ± 0.06	−0.09 ^ns^	6.32 ± 0.09	−0.04 ^ns^	6.05 ± 0.27	0.23 ^ns^	6.00 ± 0.25	0.28 ^ns^
Water control	6.28 ± 0.08	0						
(RXR)_4_XB	5.01 ± 0.14	1.34 *	4.86 ± 0.02	1.49 *	4.72 ± 0.03	1.63 *	4.58 ± 0.01	1.77 *
Water control	6.35 ± 0.13	0						

CFU, colony-forming units. The count of surviving bacteria after treatment with CPPs was compared with that of water using an unpaired *t*-test. A summary of the *p*-values is provided, with an asterisk indicating a significant difference (*p* < 0.05) and ‘ns’ denoting no significant difference.

## Data Availability

The original contributions presented in the study are included in the article, further inquiries can be directed to the corresponding author.
